# Comparing Alzheimer’s and Parkinson’s diseases networks using graph communities structure

**DOI:** 10.1186/s12918-016-0270-7

**Published:** 2016-03-02

**Authors:** Alberto Calderone, Matteo Formenti, Federica Aprea, Michele Papa, Lilia Alberghina, Anna Maria Colangelo, Paola Bertolazzi

**Affiliations:** Institute of Systems Analysis and Computer Science, National Research Council of Italy, Via dei Taurini, 19, Roma, 00185 Italy; Lab of Neuroscience “R. Levi-Montalcini”, Dept. of Biotechnology and Biosciences, University of Milano-Bicocca, Milano, 20126 Italy; Laboratory of Neuronal Networks, Department of Mental and Physical Health and Preventive Medicine, Second University of Naples, Naples, Italy, Via L. Armanni, 5, Napoli, 80138 Italy; SYSBIO Centre of Systems Biology, University of Milano-Bicocca, Milano, 20126 Italy; NeuroMI Milan Center for Neuroscience, University of Milano-Bicocca, Piazza della Scienza, 4, Milano, 20126 Italy

**Keywords:** Systems biology, Network analysis, Graphs, Alzheimer’s diseases, Parkinson’s disease, Communities, Clustering, Network comparison

## Abstract

**Background:**

Recent advances in large datasets analysis offer new insights to modern biology allowing system-level investigation of pathologies. Here we describe a novel computational method that exploits the ever-growing amount of “omics” data to shed light on Alzheimer’s and Parkinson’s diseases. Neurological disorders exhibit a huge number of molecular alterations due to a complex interplay between genetic and environmental factors. Classical reductionist approaches are focused on a few elements, providing a narrow overview of the etiopathogenic complexity of multifactorial diseases. On the other hand, high-throughput technologies allow the evaluation of many components of biological systems and their behaviors. Analysis of Parkinson’s Disease (PD) and Alzheimer’s Disease (AD) from a network perspective can highlight proteins or pathways common but differently represented that can be discriminating between the two pathological conditions, thus highlight similarities and differences.

**Results:**

In this work we propose a strategy that exploits network community structure identified with a state-of-the-art network community discovery algorithm called InfoMap, which takes advantage of information theory principles. We used two similarity measurements to quantify functional and topological similarities between the two pathologies. We built a Similarity Matrix to highlight similar communities and we analyzed statistically significant GO terms found in clustered areas of the matrix and in network communities. Our strategy allowed us to identify common known and unknown processes including DNA repair, RNA metabolism and glucose metabolism not detected with simple GO enrichment analysis. In particular, we were able to capture the connection between mitochondrial dysfunction and metabolism (glucose and glutamate/glutamine).

**Conclusions:**

This approach allows the identification of communities present in both pathologies which highlight common biological processes. Conversely, the identification of communities without any counterpart can be used to investigate processes that are characteristic of only one of the two pathologies. In general, the same strategy can be applied to compare any pair of biological networks.

**Electronic supplementary material:**

The online version of this article (doi:10.1186/s12918-016-0270-7) contains supplementary material, which is available to authorized users.

## Background

### Biological overview

Alzheimer’s disease (AD) and Parkinson’s disease (PD) are two age-related neurodegenerative diseases of the central nervous system characterized by dysfunction and death of specific neuronal populations [1, 2].

Neurological disorders exhibit a huge number of molecular alterations due to a complex interplay between genetic and environmental factors [1]. Classical reductionist approaches are focused on a few elements, providing a narrow overview of the etiopathogenic complexity of multifactorial diseases [3]. On the other hand, high-throughput technologies such as transcriptomics, proteomics, metabolomics and computational approaches allow the evaluation of many components of biological systems and their behaviors [3, 4], thus allowing for system-level investigations.

AD is the most common cause of dementia and it is characterized by progressive cognitive decline and neuronal loss accompanied by the formation of extracellular plaques of amyloid- *β* (A *β*) aggregates and intracellular neurofibrillary tangles (NTFs) of hyperphosphorylated Tau. It is also related to biochemical mechanisms, such as the unfolded protein response (UPR), mitochondrial dysfunction, neuroinflammation and vascular alterations [1].

PD is characterized by a progressive degeneration of the nigrostriatial system with loss of dopaminergic neurons in the *substantia nigra pars compacta*. Several environmental and genetic factors are correlated with PD. Among them, mutated or overexpressed *α*-synuclein aggregates impair synaptic function, affect the proteasome system and promote mitochondrial dysfunction and ROS production [2].

### Computational overview

algori One possible way of representing interaction data is using graphs (or networks). A Graph *G*=(*V*,*E*) is a mathematical object defined as a pair of sets: one set of vertices *V* (nodes, or proteins in a biological context) and one set of edges *E* (links, or interactions). *E* contains pairs (*v*_1_,*v*_2_), where *v*_1_ and *v*_2_ are contained in *V*. For instance, protein interactions can be represented as graphs, interactions between two proteins form a link between two vertices, and a whole collection of proteins and interactions forms a graph. These structures of linked entities exhibit several recurring properties and characteristics that can be used to analyze different phenomena from an holistic level, instead of using the classical reductionist approach.

Network community discovery is a procedure used to identify groups of nodes from large networks of interacting entities. These communities consist of elements connected one another that share common characteristics or features. Due to its complexity, the problem of finding communities of interconnected entities is an open problem in several disciplines varying from computer science, mathematics, and bioinformatics. These communities of interconnected entities are present in natural and, in particular, in biological networks where they represent functional modules [5]. Since it is known that the characteristics of one protein are related to the proteins sitting in its neighborhood [6], community analysis can represent a valid tool to analyze protein functions.

Generally speaking, network analysis is used to analyze biochemical pathways in larger networks [7]. As an example, the Girvan-Newman (GN) Edge Betweenness [8] algorithm is one possible approach to identify communities of nodes. This algorithm was applied to investigate how calculated communities can be used to analyze mass-spectrometry data, confirming that the community structure identified by the GN algorithm was biologically meaningful [9]. Unfortunately, since the complexity of the GN algorithm is *O*(*n*^3^), this algorithm does not scale well for large networks, implying that different algorithms need to be used.

Community discovery algorithms performances were recently compared against networks with known structure showing that a better algorithm, which outperforms GN algorithm [10], is the InfoMap [11] algorithm based on information theory principles. This algorithm is both fast and accurate for large networks with heterogeneous community sizes.

Without taking into account a network structure among interacting entities, lists of proteins or genes can be analyzed to extract common processes. More in general, comparing two pathologies exploiting lists of involved genes extracted, for instance, with some high-throughput experimental methods, is a complex and time consuming task that requires a lot of research. Entities need to be analyzed and compared, often one by one, in order to understand common and different characteristics. Alternatively, the analysis of large lists of genes can be done automatically using DAVID, which also assigns a significance value (*p*-value) to characteristic terms [12].

Comparative approaches were also useful to identify cancer-specific gene signatures [13] and the relevance of metabolism in human cancer [14, 15], as well as to investigate networks and genes linking sleep and stress disturbances in neuropsychiatric disorders [16].

### Strategy description

**Fig. 1:**
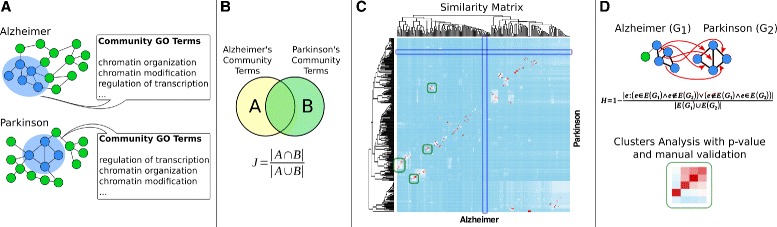
Experimental design. **a** Starting from the two induced networks, communities were calculated (blue circles) and for each of them a list of Gene Ontology terms was retrieved. **b** Communities term lists were compared calculating Jaccard similarity, which was then reported in a similarity matrix (red high overlap, blue low overlap). **c** The similarity matrix consists of communities that contain significant terms (Benjamini *p*-value <0.05). A clustering algorithm revealed areas (green squares) that represent common processes, while communities without any high overlap counterpart (blue long rectangles) were analyzed to find specific processes of the two pathologies **d**) Network topology was analyzed to assess structure overlap between pairs (Hamming distance) of communities concluding that topology implies biological process but not vice-versa. Clustered green areas were further analyzed by assigning to terms in the clusters a significance *p*-value

In this work we propose a new strategy that exploits network community structure identified with InfoMap in order to compare two similar and yet different pathologies AD [17] and PD [18]. We introduce a graph-communities-based Similarity Matrix that can be used to cross-compare two pathologies in order to highlight similarities and differences in terms of functions and network topology. Communities present in both pathologies can be analyzed to highlight common biological processes. Conversely, communities without any counterpart are used to investigate processes that are characteristic of each of the two pathologies separately. Figure 1 summarizes the entire approach. Datasets supporting the results of this article are included in Additional file 1.

## Results and discussion

To compare AD and PD from a network perspective, we took the two starting lists of AD [17] and PD [18] proteins without considering network structure and we enriched them with Gene Ontology terms describing biological processes. We obtained 827 significant Gene Ontology terms from AD list, and 550 terms from PD list. The simple intersection between these two lists resulted in 368 common terms, which was large and hard to evaluate. Despite this richness of terms, known processes involved in both pathologies, such as RNA splicing, histone modification, DNA repair and others, either were missing or had not significant *p*-values, suggesting that a more refined analysis was needed.

Using the two starting lists, we derived two networks from the human interactome [19]. We found that both networks were compliant with what are proposed to be natural networks [20]. Both starting networks were small-world, scale-free [8, 21] and ultra small [22] with an average path length in the order of *ln*(*ln*(*N*)), where *N* is the number of nodes in the network. Table 1 summarizes this analysis.

**Table 1:** Networks characteristics and metrics

	Alzheimer’s	Parkinson’s	Influenza	mTOR		
	disease	disease				
Seed nodes	302	454	176	2362		
Induced graph nodes	5,262	6,051	4,010	8,009		
Induced graph edges	20,205	22,296	16,632	25,812		
Average degree	7.680	7.369	8.295	6.446		
Average path length	3.013	3.031	2.841	3.244		
*Small-World*	*8.57*	*8.708*	*8.296*	*8.988*		
*Ultra Small*	*2.148*	*2.162*	*2.116*	*2.196*		
Power-law exponent	2.885	2.831	1.743	1.509		
Average transitivity	0.013	0.011	0.015	0.008		
InfoMap communities	372	422	227	572		

### Preliminary networks comparison

As shown in Table 2A, this preliminary analysis confirmed that AD and PD networks have good similarities both in terms of entities involved [12 %, which was higher than Influenza (8 %) and mTOR (6 and 8 % versus AD and PD, respectively)], and in terms of links contained in the induced graphs (81 % of edges in common). Indeed, by observing these measurements (Table 2B), we concluded that AD and PD are more similar to each other in terms of networks structure (81 %), than they are to Influenza (69 and 68 % versus AD and PD, respectively). A greater distance would not be reasonable, as both neuropathologies and Influenza share inflammatory responses. Likewise, Table 2A and B show that both AD and PD share entities (6 and 8 % versus AD and PD, respectively) and interactions (77 and 86 %, respectively) with the mTOR pathway, because of the central role of mTOR in regulating neuronal homeostasis in response metabolic and energy requirements, as well as in influencing neuronal function and synaptic plasticity [23]. Moreover, inhibition of mTOR signaling plays an essential role in neuroprotection by clearing aggregated proteins and dysfunctional mitochondria in these and other neurodegenerative conditions [23]. These considerations were also confirmed by data in Table 2C, where we calculated the amount of common communities with GO terms similarity within the first and fifth quintile. Not surprisingly, all networks overlapped and, as expected, mTOR had a good overlap with both the neurodegenerative diseases at study. This result is also a consequence of the vastness of the mTOR map analyzed, which contained more than 2300 different proteins resulting in an induced graph with more than 8000 nodes and more than 25,000 edges (see Table 1). On the other hand, it would be very difficult to find a biological network without overlaps with AD/PD, as these neuropathologies are often associated with co-morbidities. Moreover, neuronal degeneration also involves activation of cell cycle events (see Additional file 2), which might be considered as peculiar of cancer growth.

**Table 2:** Entities, networks and communities overlap comparisons

A) Common entities					
	Alzheimer				
Alzheimer	-	Parkinson			
Parkinson	12 %	-	Influenza		
Influenza	8 %	8 %	-	mTOR	
mTOR	6 %	8 %	3 %	-	Random*
Random*	0.17 %	0.11 %	0.28 %	0.02 %	-
B) Common interactions					
	Alzheimer				
Alzheimer	-	Parkinson			
Parkinson	81 %	-	Influenza		
Influenza	69 %	68 %	-	mTOR	
mTOR	77 %	86 %	64 %	-	Random*
Random*	8.83 %	7.7 %	8.97 %	3.5 %	-
C) Similar communities					
	Alzheimer				
Alzheimer	-	Parkinson			
Parkinson	36 %	-	Influenza		
Influenza	28 %	27 %	-	mTOR	
mTOR	35 %	39 %	22 %	-	Random*
Random*	0.66 %	1.18 %	0.15 %	2.47 %	-

A) shows the percentage of common entities among the four lists analyzed calculated with Jaccard distance. B) Shows the overlap in terms of links between the four induced networks analyzed calculated with Hamming similarity. C) shows results obtained counting overlapping community pairs that have a functional similarity that falls in the fifth quintile. (*) Values calculated by averaging the results obtained against 100 randomly generated sets of comparable sizes

### Considerations about signaling networks

Signaling networks, despite being different from PPI networks, may provide useful information to analyze communities that exert signaling functions. Even though PPI imply physical contacts while signaling interactions are often “long range” interactions, which hampers the automatic merge of these two kinds of networks, we partially analyzed the largest published signaling networks [24].

Table 3 shows that the coverage of the utilized signaling network is good but lower than the one of the *mentha* PPI network. Furthermore, among all the entities included in the analyzed signaling networks, we calculated that 92 % were also contained in *mentha*. Finally, since signaling networks currently do not provide interaction reliability scores, we could not perform the proposed method. In our case the InfoMap [11] network community discovery algorithm needs scored interactions.

**Table 3:** Comparison with signaling networks. Protein-protein interaction networks currently have an higher coverage than signaling networks

	Seed proteins in network			
	Alzheimer	Parkinson	Influenza	mTOR
mentha (PPI)	99 %	100 %	91 %	98 %
Zaman et al. (Signaling)	87 %	76 %	82 %	73 %

These considerations do not rule out that an analysis similar to the one proposed in our work might be performed again in the near future, as these networks grown in coverage and curation detail, hopefully with the aid of a common curation policy that might also help data integration, like it happened for protein interaction networks [25].

We refined the basic Gene Ontology analysis by subdividing the starting network into communities obtaining 372 communities for AD and 422 communities for PD. We used these communities to analyze similarities in terms of biological processes and network topology. By enriching each community with Gene Ontology terms, we created lists of biological processes that describe each identified group. Only communities containing terms with a significant Benjamini corrected *p*-value (*p*-value ≤0.05) were retained, thus reducing the number of analyzed communities from 372 to 186 in AD, and from 422 to 222 in PD.

Instead of manually going through 186×222 pairs to find relevant terms, we used a Similarity Matrix to perform a clustering algorithm in order to identify areas to investigate.

**Fig. 2:**
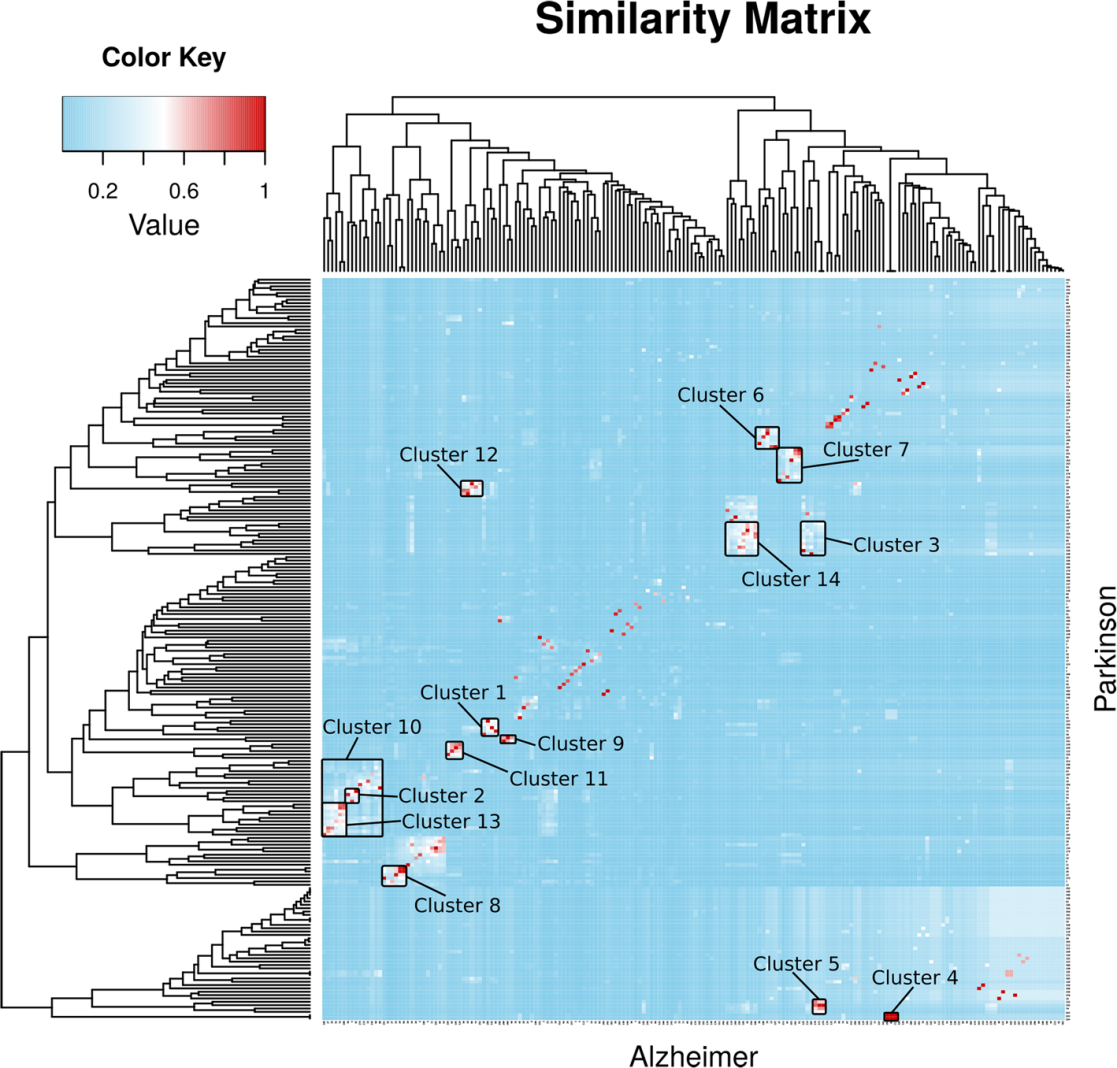
Similarity matrix. This matrix shows statistically significant communities found in Alzheimer’s and Parkinson’s diseases protein-protein interaction networks clustered according to their Gene Ontology overlap. Green areas are clusters that might reveal strong significance. Single red dots are communities that are almost exclusively overlapped between the two pathologies

Starting from the results obtained from the computational strategy, we performed two analyses. First, we investigated pairs of communities that had a similarity within the fifth quintile of the similarity distribution and well clustered areas identified on the Similarity Matrix (Fig. 2). This findings allowed us to conclude that most of the biological processes involved in AD and PD are similar, which is in compliance with the fact that AD and PD are both neurodegenerative diseases. Furthermore, we were able to identify processes such as DNA repair, RNA metabolism and glucose metabolism that were not detected by simple Gene Ontology Enrichment analysis. Second, by analyzing communities with similarity within the first quintile, we identified 10 communities in PD and 8 communities in AD that contained specific processes for the two pathologies (Table 4). It is worth mentioning that this approach also highlighted not yet clarified phenomena that will be considered for our future studies and promote new working hypotheses.

**Table 4:** Specific processes for AD and PD. List of processes that do not have a counterpart in both pathologies

Alzheimer’s disease		Parkinson’s disease	
Community	Description	Community	Description
33	Cell motility and adhesion	96	Blood vessel development
135	Lipid metabolism and transport	109	Glutamatergic synaptic transmission
163	PDGF signaling pathway	150	TGF signaling pathway
174	Tetrahydrobiopterin biosynthesis	164	Synaptic vesicles secretion
175	IGF signaling pathway	169	Dopaminergic transmission
243	IL6 and CNTF signaling pathway	179	FGF signaling pathway
330	Blood coagulation	185	Purine/pyrimidine metabolism
365	Endothelin signaling pathway	323	Chemotaxis
		364	Proteoglycan biosynthesis
		385	Inner mitochondrial membrane organization

For instance, we found that community 174 of AD includes enzymes catalyzing the synthesis of tetrahydropterin (BH4). In addition to its role as a cofactor in the biosynthesis of monoamine neurotransmitters (adrenaline/noradrenaline, dopamine and serotonin) and in the balance of nitric oxide, BH4 is also an important regulator of the cellular redox state by shuttling reducing equivalents from NADPH to specific substrates. More studies will be also needed to elucidate the significance of PDGF or collagen (community 163) in AD, as well as the relevance of FGF (community 179) in PD, most likely for their role in neurogenesis and angiogenesis. Finally, community 185 in AD is particularly interesting as its terms are related to the biosynthesis of purine and pyrimidine, which is something poorly investigated. The entire list of identified communities is available in supplementary data (Additional files 2 and 3).

Using significantly functional communities, we also investigated which communities actually had a similar topology and which communities, despite their functional similarity, had different topologies. In accordance with the known relationship between communities and biological functions, we did not find any community with high topological similarity and low Gene Ontology similarity, suggesting that topology implies biological processes but not vice-versa. This is not surprising as various sets of proteins can exert similar biological processes, such as transcription regulation, stress response and so on.

Our InfoMap based computational strategy, while confirming the relevance of the PD-map by Fujita [18], provided a new tool to capture the potential connection between neuronal mitochondrial dysfunction, glucose metabolism and glutamate/glutamine cycle (which also involve astroglial responses), as recently implemented in the on-line PD map [18]. This finding strengthens the need for detailed metabolomic studies.

## Conclusions

In conclusion, understanding neurodegenerative diseases is a task that requires different strategies and approaches. By using a community discovery algorithm based on information theory principles and by using two community-wise similarity measurements, we were able to identify communities of proteins that describe processes involved in two distinctive and yet similar pathologies. Overall, our approach can be used to compare any pair of biological networks. In particular, we identified similarities and differences between AD and PD, which can in turn promote cross-seeding between groups working on the two pathologies separately.

## Methods

All datasets used in this work were publically available and we did not require any ethic approval to access and use them.

### Networks comparison

To start our analysis, we collected genes and proteins from two SBML models describing AD [17] and PD [18] and complemented these two lists with data downloaded from the KEGG database [26]. AD list contained 302 proteins while PD list contained 454 proteins.

Direct comparison of SBML models is not feasible due to subjectivity: biochemical reactions can be described at different level of detail and with different entities and terminology. Therefore, we moved our analysis on the human interactome [19] and, by using these “seed proteins”, we extracted two subnetworks, one for AD and one for PD.

At first, we assessed what was in common between the starting lists of proteins and their respective induced graphs extracted from the entire human interactome. At the same time, we assessed whether AD and PD networks were actually closer to each other than they were to other potentially unrelated networks. To this end, we compared AD and PD networks against another large SBML model describing Influenza [27] and a large SBML model describing the mTOR pathway [28]. All models were processed in the same way, as described in *Networks Assembly and Validation*. The comparison between AD and PD against these two models is justified by the fact that all four models are large enough to be comparable. Several smaller models are available [29] but they are not as comprehensive as those considered in this work.

We calculated Jaccard similarity [30] (Common entities over all entities) between the two starting lists and Hamming distances [31, 32] (Common edges) between the two starting networks. Details about these measurements are reported in *Similarity Measurements*.

### Networks assembly and validation

To uniform data extracted from SBML models and KEGG, we translated all proteins and genes to UniProt [33] Accession Numbers using UniProt mapping API. This allowed us to extract protein-protein interaction networks from the *mentha* [19] weighted human interactome, a free database that offers ready-to-use merged data from different resources (namely IntAct [25, 34], MINT [35], DIP [36], MatrixDB [37] and BioGrid [38]). *mentha* uses the same data curation policy promoted by the IMEx consortium, granting for a manual-quality interaction network.

**Fig. 3:**
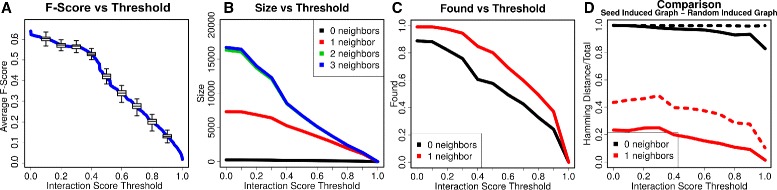
Interactions filtering threshold. **a** F-Score against Reactome. 100-Fold validation. Averaged F-Score decreases after a cutoff of 0.4 suggesting that any threshold greater than 0.4 would lose Reactome’s interactions. **b** Network Expansion. Induced graph expansion on a starting set of about 400 vertices. By taking neighbors at distance two or three from seed nodes we captured almost the entire human interactome suggesting that the best choice was taking only the first neighbors. **c** Recall. Average fraction of seed proteins captured in both networks at each threshold. **d** Similarity between networks and random networks. Dashed lines show distance from random networks, continuous lines show distance between AD and PD networks. Distance 0, identical networks; distance 1, completely different networks. Difference between analyzed networks was of about 20 % at threshold 0.4, which was lower than the difference between these networks and random networks (40 %) suggesting the two networks at study are similar

Since interactions archived in *mentha* are weighted, we chose a filtering threshold to reduce false positives. We performed three analysis: *F-Score*, *Network Expansion*, *Seed Proteins Recall* (Fig. 3).

First of all, we wanted to find a filtering threshold that could approximate the functional information archived in Reactome [39], a well-established pathway database that contains data similar to those of the biological models used in our starting datasets (i.e. biochemical reactions). We used Reactome as a positive set (152,267 interactions) and added a ten times larger set of random interactions not present in Reactome (negatives). To this end, we calculated the best *F-Score* (the harmonic mean of precision and recall). We performed a 100-fold validation to analyze how *mentha* scores approximated Reactome interactions as cutoff changes. Figure 3a shows how *F-Score* starts to substantially decrease after a cutoff of 0.4.

Since we had to extract subgraphs from the entire human interactome, we also wanted to be sure that the induced graphs were not too large, to prevent computational problems and to minimize the amount of noise introduced in the analysis. Using “seed proteins”, we investigated how large the induced graph became with respect to the “neighborhood radius” - i.e., if we take only the first neighbors or also neighbors of neighbors and so on. We wanted that our induced networks were large enough to capture the information needed to define communities without degenerating into a too large network. From this second analysis, we concluded that taking the first neighbors of each seed protein was a fair choice to control *Network Expansion*, Fig. 3b. We concluded that an edge score threshold of 0.4 and a neighborhood radius of 1 was the best choice.

Finally, we wanted to be sure that we had the best *Seed Proteins Recall* possible so that most of the starting proteins were actually included in the induced graphs. To verify this, we counted how many seed proteins were contained in the induced graphs. Figure 3c shows that a threshold of 0.4/0.5 captured more than 80 % of the seed proteins, justifying once again the chosen threshold and neighborhood radius.

Having these two networks, we wanted to verify that they are dissimilar to random networks but similar to each other, justifying their comparison. First of all, we quantified the actual difference between these induced graphs and random graphs generated from comparable random seed protein sets. Secondly, we calculated how AD and PD networks are similar to each other. To calculate graphs similarity, we used the H distance. Using this distance, we confirmed that with a threshold of 0.4 and a neighborhood radius of 1 we obtained networks that are distant from random networks but similar to each other, Fig. 3d.

### Similarity measurements

Throughout our study, we used two similarity measurements, one that measures entities overlap (Genes, Proteins, Gene Ontology terms), and one that considers network structure. We computed Jaccard similarity (J) [30] to quantify the ratio of the intersection of two sets over their union. We calculated the complement of the Hamming distance (H) [31, 32] for network topology; this second measurement is similar to Jaccard similarity, but it considers different network links (*e*:(*e*∈*E*(*G*), where *e* is a link and *G* is a graph) instead of common entities.

(1) $$ {\selectfont{\begin{aligned} &{}J=\frac{|A\cap B|}{|A\cup B|} \\ &{}H=1-\frac{|e:(e\in E(G_{1})\wedge e\notin E(G_{2}))\vee (e\notin E(G_{1})\wedge e\in E(G_{2}))|}{|E(G_{1})\cup E(G_{2})|} \end{aligned}}}  $$

### Communities and similarity matrix analysis

We divided the two starting networks in communities to highlight areas that exert specific functions in the two pathologies. To extract communities – i.e. relevant interconnected subareas of a network – we used the InfoMap [11] algorithm which, as shown by Lancichinetti and Fortunato, has good performances on networks characterized by heterogeneous community sizes and degree distributions [10]. InfoMap algorithm works by assigning strings of bits to each node in the network. These bits are assigned in ways that describe nodes organized in groups of strongly interconnected entities. The algorithm minimizes the number of bits needed to describe network structure.

After network communities were identified, we wanted to analyze them from a biological process perspective. To assign a biological meaning to each community, we performed Gene Ontology enrichment at lower levels “FAT” by using the RDAVIDWebService [40] Bioconductor [40–42] package. This kind of analysis allowed us to automatically collect processes involved in the two neurodegenerative diseases at study. These pathologies are the result of a great variety of pathways and processes that are hard to enumerate without an automatic procedure like Gene Ontology Terms enrichment. In general, Gene Ontology Enrichment labels entities with a series of terms that are then statistically ranked according to their abundance. This approach allowed us to assign to each community a list of terms with their respective *p*-values. By taking into account significance values with Benjamini correction [43], only communities with statistically relevant terms were analyzed. To find similar communities and different ones, we compared network topology and terms assigned to each community.

We calculated pairwise J similarity for terms and pairwise H distance for subnetworks. J similarity was used to construct a Similarity Matrix (Fig. 2) that was then clustered using euclidean distance. This clustering step revealed areas in the Similarity Matrix that were statistically evaluated, assigning to each term in the clusters a *p*-value calculated with respect to the entire Similarity Matrix. This calculation was performed by creating, for each cluster, 10,000 random sets with the same terms distribution as the entire matrix. This last step allowed us to identify statistically significant processes contained in the clusters identified in the Similarity Matrix.

Finally, while common processes were identified through community dissection and clustering, distinctive processes associated to the two pathologies were extracted from the Similarity Matrix by scanning rows and columns retaining communities with similarity within the first quintile in order to find communities with no relevant counterpart in the other pathology.

## Availability of data and materials

The dataset(s) supporting the conclusions of this article is (are) available in the FigShare repository https://dx.doi.org/10.6084/m9.figshare.2070124.

## Additional files

Additional file 1 Datasets: communities, networks, enrichments (ZIP available at https://dx.doi.org/10.6084/m9.figshare.2070124).

Additional file 2 Common Processes. (PDF 66 kb)

Additional file 3 Pathology Specific Processes. (PDF 66 kb)

## References

[1] Alberghina L, Colangelo AM (2006). The modular systems biology approach to investigate the control of apoptosis in Alzheimer’s disease neurodegeneration. BMC Neurosci.

[2] Jenner P, Morris HR, Robbins TW, Goedert M, Hardy J, Ben-Shlomo Y, Bolam P, Burn D, Hindle JV, Brooks D (2013). Parkinson’s disease–the debate on the clinical phenomenology, aetiology, pathology and pathogenesis. J Park Dis.

[3] Minguez P, Parca L, Diella F, Mende DR, Kumar R, Helmer-Citterich M, Gavin AC, van Noort V, Bork P (2012). Deciphering a global network of functionally associated post-translational modifications. Mol Syst Biol.

[4] Barabási AL, Oltvai ZN (2004). Network biology: understanding the cell’s functional organization. Nat Rev Genet.

[5] Spirin V, Mirny LA (2003). Protein complexes and functional modules in molecular networks. Proc Natl Acad Sci U S A.

[6] Schwikowski B, Uetz P, Fields S (2000). A network of protein-protein interactions in yeast. Nat Biotechnol.

[7] Holme P, Huss M, Jeong H (2003). Subnetwork hierarchies of biochemical pathways. Bioinformatics (Oxford, England).

[8] Girvan M, Newman MEJ (2002). Community structure in social and biological networks. Proc Natl Acad Sci U S A.

[9] Dunn R, Dudbridge F, Sanderson CM (2005). The use of edge-betweenness clustering to investigate biological function in protein interaction networks. BMC Bioinforma.

[10] Lancichinetti A, Fortunato S (2009). Community detection algorithms: a comparative analysis. Phys Rev E.

[11] Rosvall M, Axelsson D, Bergstrom CT (2010). The map equation. Eur Phys J Spec Top.

[12] Huang DW, Sherman BT, Lempicki RA (2009). Systematic and integrative analysis of large gene lists using DAVID bioinformatics resources. Nat Protoc.

[13] Balestrieri C, Vanoni M, Hautaniemi S, Alberghina L, Chiaradonna F. Integrative transcriptional analysis between human and mouse cancer cells provides a common set of transformation associated genes. Biotechnol Adv; 30(1):16–29. doi:http://dx.doi.org/10.1016/j.biotechadv.2011.06.013. 10.1016/j.biotechadv.2011.06.013 21736933

[14] Gaglio D, Metallo CM, Gameiro PA, Hiller K, Danna LS, Balestrieri C, Alberghina L, Stephanopoulos G, Chiaradonna F (2011). Oncogenic K-Ras decouples glucose and glutamine metabolism to support cancer cell growth. Mol Syst Biol.

[15] Alberghina L, Gaglio D (2014). Redox control of glutamine utilization in cancer. Cell Death Dis.

[16] Jiang P, Scarpa J, Fitzpatrick K, Losic B, Gao V, Hao K, Summa K, Yang H, Zhang B, Allada R, Vitaterna M, Turek F, Kasarskis A (2015). A systems approach identifies networks and genes linking sleep and stress: implications for neuropsychiatric disorders. Cell Rep.

[17] Mizuno S, Iijima R, Ogishima S, Kikuchi M, Matsuoka Y, Ghosh S, Miyamoto T, Miyashita A, Kuwano R, Tanaka H (2012). AlzPathway: a comprehensive map of signaling pathways of Alzheimer’s disease. BMC Syst Biol.

[18] Fujita Ka, Ostaszewski M, Matsuoka Y, Ghosh S, Glaab E, Trefois C, Crespo I, Perumal TM, Jurkowski W, Antony PMa, Diederich N, Buttini M, Kodama A, Satagopam VP, Eifes S, Del Sol A, Schneider R, Kitano H, Balling R (2014). Integrating pathways of Parkinson’s disease in a molecular interaction map. Mol Neurobiol.

[19] Calderone A, Castagnoli L, Cesareni G (2013). Mentha: a resource for browsing integrated protein-interaction networks. Nat Methods.

[20] Jeong H, Albert R, Tombor B, Oltvai ZN, Barabási AL (2000). The large-scale organization of metabolic networks. Nature.

[21] Newman MEJ (2003). The structure and function of complex networks. SIAM Rev.

[22] Cohen R, Havlin S (2003). Scale-free networks are ultrasmall. Phys Rev Lett.

[23] Nixon RA (2013). The role of autophagy in neurodegenerative disease. Nat Med.

[24] Zaman N, Li L, Jaramillo M, Sun Z, Tibiche C, Banville M, Collins C, Trifiro M, Paliouras M, Nantel A, O’Connor-McCourt M, Wang E (2013). Signaling network assessment of mutations and copy number variations predict breast cancer subtype-specific drug targets. Cell Rep.

[25] Orchard S, Ammari M, Aranda B, Breuza L, Briganti L, Broackes-Carter F, Campbell NH, Chavali G, Chen C, Del-Toro N, Duesbury M, Dumousseau M, Galeota E, Hinz U, Iannuccelli M, Jagannathan S, Jimenez R, Khadake J, Lagreid A, Licata L, Lovering RC, Meldal B, Melidoni AN, Milagros M, Peluso D, Perfetto L, Porras P, Raghunath A, Ricard-Blum S, Roechert B, Stutz A, Tognolli M, van Roey K, Cesareni G, Hermjakob H (2014). The MIntAct project–IntAct as a common curation platform for 11 molecular interaction databases. Nucleic Acids Res.

[26] Kanehisa M, Goto S (2000). KEGG: Kyoto encyclopedia of genes and genomes. Nucleic Acids Res.

[27] Matsuoka Y, Matsumae H, Katoh M, Eisfeld AJ, Neumann G, Hase T, Ghosh S, Shoemaker JE, Lopes TJS, Watanabe T, Watanabe S, Fukuyama S, Kitano H, Kawaoka Y (2013). A comprehensive map of the influenza A virus replication cycle. BMC Syst Biol.

[28] Caron E, Ghosh S, Matsuoka Y, Ashton-Beaucage D, Therrien M, Lemieux S, Perreault C, Roux PP, Kitano H (2010). A comprehensive map of the mTOR signaling network. Mol Syst Biol.

[29] Juty N, Ali R, Glont M, Keating S, Rodriguez N, Swat M, Wimalaratne S, Hermjakob H, Le Novère N, Laibe C, Chelliah V (2015). BioModels: content, features, functionality, and use. CPT: Pharmacometrics Syst Pharmacol.

[30] Levandowsky M, Winter D (1971). Distance between Sets. Nature.

[31] Butts CT, Carley KM. Multivariate methods for interstructural analysis; 2001. CASOS working paper, Center for the Computational Analysis of Social and Organisation Systems, Carnegie Mellon University, http://www.casos.cs.cmu.edu/publications/papers/multiv001a.pdf.

[32] Hamming RW (1950). Error detecting and error correcting codes. Bell Syst Tech J.

[33] Magrane M, Consortium UP. UniProt Knowledgebase: a hub of integrated protein data. Database. 2011; 2011:009–009. http://www.ncbi.nlm.nih.gov/pubmed/26896845. 10.1093/database/bar009 PMC3070428 21447597

[34] Kerrien S, Aranda B, Breuza L, Bridge A, Broackes-Carter F, Chen C, Duesbury M, Dumousseau M, Feuermann M, Hinz U, Jandrasits C, Jimenez RC, Khadake J, Mahadevan U, Masson P, Pedruzzi I, Pfeiffenberger E, Porras P, Raghunath A, Roechert B, Orchard S, Hermjakob H (2012). The IntAct molecular interaction database in 2012. Nucleic Acids Res.

[35] Licata L, Briganti L, Peluso D, Perfetto L, Iannuccelli M, Galeota E, Sacco F, Palma A, Nardozza AP, Santonico E, Castagnoli L, Cesareni G (2012). MINT, the molecular interaction database: 2012 update. Nucleic Acids Res.

[36] Salwinski L, Miller CS, Smith AJ, Pettit FK, Bowie JU, Eisenberg D (2004). The Database of Interacting Proteins: 2004 update. Nucleic Acids Res.

[37] Chautard E, Fatoux-Ardore M, Ballut L, Thierry-Mieg N, Ricard-Blum S (2011). MatrixDB, the extracellular matrix interaction database. Nucleic Acids Res.

[38] Chatr-Aryamontri A, Breitkreutz BJ, Heinicke S, Boucher L, Winter A, Stark C, Nixon J, Ramage L, Kolas N, O’Donnell L, Reguly T, Breitkreutz A, Sellam A, Chen D, Chang C, Rust J, Livstone M, Oughtred R, Dolinski K, Tyers M (2013). The BioGRID interaction database: 2013 update. Nucleic Acids Res.

[39] Matthews L, Gopinath G, Gillespie M, Caudy M, Croft D, de Bono B, Garapati P, Hemish J, Hermjakob H, Jassal B, Kanapin A, Lewis S, Mahajan S, May B, Schmidt E, Vastrik I, Wu G, Birney E, Stein L, D’Eustachio P (2009). Reactome knowledgebase of human biological pathways and processes. Nucleic Acids Res.

[40] Fresno C, Fernández EA (2013). RDAVIDWebService: a versatile R interface to DAVID. Bioinformatics (Oxford, England).

[41] Gentleman RC, Carey VJ, Bates DM, Bolstad B, Dettling M, Dudoit S, Ellis B, Gautier L, Ge Y, Gentry J, Hornik K, Hothorn T, Huber W, Iacus S, Irizarry R, Leisch F, Li C, Maechler M, Rossini AJ, Sawitzki G, Smith C, Smyth G, Tierney L, Yang JYH, Zhang J (2004). Bioconductor: open software development for computational biology and bioinformatics. Genome Biol.

[42] Huber W, Carey VJ, Gentleman R, Anders S, Carlson M, Carvalho BS, Bravo HC, Davis S, Gatto L, Girke T, Gottardo R, Hahne F, Hansen KD, Irizarry RA, Lawrence M, Love MI, MacDonald J, Obenchain V, Oleś AK, Pagès H, Reyes A, Shannon P, Smyth GK, Tenenbaum D, Waldron L, Morgan M (2015). Orchestrating high-throughput genomic analysis with Bioconductor. Nat Methods.

[43] Benjamini Y, Hochberg Y (1995). Controlling the false discovery rate: a practical and powerful approach to multiple testing. J R Stat Soc Ser B Methodol.

