# Supplementary material for: Comparing Alzheimer’s and Parkinson’s diseases networks using graph communities structure
Source: BMC Syst Biol. 2016 Mar 2;10:25. doi: 10.1186/s12918-016-0270-7 (PMC4776441; doi:10.1186/s12918-016-0270-7)
Supplement: Additional file 2 — Common Processes. (PDF 66 kb) [file 12918_2016_270_MOESM2_ESM.pdf]

Additional File 2 – Alzheimer's and Parkinson's Diseases Common Processes

Here we described common biological processes that characterize the two neurodegenerative diseases. These processes were identified by analyzing clustered areas in the Similarity Matrix (Figure 1, main article) and by computing, for each of the terms captured in these areas, an empirical p-value against 10,000 random sets. As a result, some relevant processes, not present in the simple Gene Ontology enrichment analysis, emerged. We reviewed some of these processes and reported references that support these findings.

Glucose Metabolism and Phosphate Metabolism Processes

The brain is the organ with the highest energy demand. Although the brain represents only about 2% of total body weight, approximately 20% of oxygen and 25% of glucose consumed by the human body are intended to brain function[1]. Maintaining and restoring ionic gradients following synaptic transmission, as well as neurotransmitters internalization and recycling, are processes that require a lot of energy. Since neuronal metabolism is purely oxidative, there is a close correlation between metabolism and vascularization[2].

Glucose metabolism is crucial for neuron viability and function, thus its decrease plays a critical role in many neurological diseases. Bioenergetics and mitochondrial defects have long been proposed as the mechanisms underlying chronic neuronal dysfunction and death. Indeed, AD patients exhibit reduced glucose energy metabolism, even at an early stage of the disease[2,3]. Therefore, O_2 consumption, glucose and blood flow are used to evaluate brain function by positron emission tomography (PET) using the radioactive glucose analogue ^{18}Fluoro-2-deoxyglucose. PET has been used to track AD-related dysfunction by estimating glucose utilization rate[1]. Changes in glucose metabolism could be caused by a reduction of glucose uptake through glucose transporters, mitochondrial dysfunction or changes in mitochondrial dynamics. Also familial PD, reproduced in α-synuclein transgenic PD models, displays impaired mitochondrial function and metabolism. In addition, mutations in other PD-related proteins (such as PINK1, parkin and DJ-1) are involved in the regulation of mitochondrial function, which are also the target of all PD-related toxins (rotenone, paraquat, etc)[4].

These processes have significant p-values in clusters 8, 9 and 11.

ATP production is the main goal of cell metabolism, and used as an energy supplier or as a donor of phosphate groups. Many intracellular signals operate with an on/off mechanism based on protein phosphorylation mediated by kinases[4–6].

These processes have significant p-values in clusters 6, 10, 11 and 13.

DNA Damage, Apoptosis and Cell Cycle

The genome is constantly exposed to endogenous and exogenous genotoxic agents. Oxidative damage to the DNA is particularly harmful: over 100 oxidative-based modifications have been identified and these alterations could be highly mutagenic, while others block replication or transcription. Many studies have shown that ROS are a major source of DNA damage in the brain due to its highly oxidative metabolism. Genomic stability requires a number of biochemical pathways involving different proteins and processes that lead to DNA repair. The correlation between defects in DNA damage repair and brain disorders is well documented: mutations in ATM, a protein implicated in non-homologous end joining (NHEJ) pathways, are responsible for ataxia-telangiectasia (AT); mutation in proteins involved in nucleotide excision repair (NER) cause the Cockayne Syndrome (CS), in which neurodegeneration is included among clinical features; base excision repair (BER) has evolved to resolve base modification, and the decreased activity of this mechanism has been correlated with age-related neurogenerative disorders, such as AD and PD[7–10].

Excessive accumulation of DNA damage or lack of repair mechanisms induce apoptosis as a strategy to prevent damages to neighboring cells. This could be an advantage to ensure tissue homeostasis and turnover, but neurons in adult organisms cannot be replaced. DNA damage, as well

as other cellular stresses (ER stress, mitochondrial dysfunction, UPS dysfunction, inflammation, etc.) can trigger apoptosis, a finely regulated process that is performed by proteolytic enzymes called caspases[11].

Programmed cell death is crucial for normal neural development. It regulates the number and types of cells in the developing brain and it plays a key role in constructing proper target innervation and neuronal networks. Under pathologic conditions, it is responsible for neuronal loss in neurodegenerative diseases, as well as in physiologic aging[11–14]. Mechanisms of neuronal degeneration in AD are still largely unclear. Toxic effects of Aβ is the consequence of ROS production and apoptosis induction. In this regard, in vivo and in vitro experiments have shown that soluble Aβ impairs mitochondria metabolism by decreasing cytochrome oxidase activity and increasing hydrogen peroxide generation[12]. ER stress has been reported as another causative factor in AD by affecting tau phosphorylation and Ca^{2+} regulation. Epidemiological studies have introduced several factors that increase susceptibility to PD including pesticides, herbicides and industrial chemicals. These substances promote mitochondrial dysfunction and release of cytochrome c from mitochondria as crucial events of intrinsic apoptosis[14].

Apoptotic neuronal death is also the result of abortive cell cycle: following toxic insults, neurons start to divide[15,16]. Cell cycle involves the coordination of three distinct processes: mass accumulation, DNA replication and partition of the genetic material that leads to cell duplication and separation. This process is conserved for the majority of cell types. Neurons, however, are able to start the replication process, but they are not able to divide. Instead of going to mitosis, neurons that re-enter cell cycle die by apoptosis. The block of cell cycle is fundamental in adult neurons. Neurons do not die, nor they can complete the cell cycle (no evidence of M-phase has ever been reported). Neurons of adult mice or humans CNS can exist in this abnormal 'hyperploid' state for months or years. A number of studies have shown the presence of active cell cycle proteins and complexes in neurons of AD brain, including cyclins (Cyclin D and E), cell cycle kinases (cdk2 and cdk4), as well as their activators and inhibitors (p27, p19, Ki67, etc), which are indicative of cell cycle re-entry. A typical apoptotic process takes only 12 hours to complete, thus death by cell cycle in adult neurons seems to be a very slow process. This protracted time is unexpected[12,17,18].

DNA damage and repair processes have significant p-values in clusters 7, 8, 10, 11 and 13.

Cell cycle processes have significant p-values in clusters 1, 6, 7, 8, 10, 11 and 13.

Apoptosis components have significant p-values in clusters 4 and 8.

Protein Localization and Vesicles Trafficking

Once synthesized, proteins need to be correctly localized in order to exert their biological function. Protein trafficking is finely regulated at different levels: from signal sequences to post-translational modifications or storage in endosomes and vesicles for extracellular transport.

In this regard, neuronal cells are specialized in vesicular transport, which can be anterograde (along the axon) or retrograde (towards the soma), for neurotransmitters release and re-uptake during synaptic activity. This process affects differently both diseases. For instance, retrograde transport of NGF (the neurotrophin designed for the survival of cholinergic neurons) is altered in AD, while α-synuclein mutations seems to affect the release of dopamine vesicles in PD[19,20].

These processes have significant p-values in cluster 1, 3, 5, 11 and 14

RNA Metabolism and Regulation of Transcription

RNA synthesis and maturation are common and essential for any cells. To give a detailed description of mechanisms linked to RNA metabolism and neurodegenerative diseases goes beyond the scope of this work. It is known that various RNA metabolism events are altered in complex and multifactorial pathologies. In general, diseases with altered RNA processing are defined as "RNApathies". In some neurological diseases it is clear the correlation between aberrant RNA mechanisms and phenotype, while in others the correlation is much more elusive, as in the case of neurodegeneration[21–24].

RNA toxicity occurs through multiple mechanisms and at multiple levels: the expansion of triplets in a gene may result in a gain of function and the formation of nuclear RNA foci that lead to haplo-insufficiency or to the production of proteins with multiple poly-aminoacids (poly-Q, poly-A, poly-D, …) which may result as non-functional or even toxic. Furthermore, aberrant splicing, bidirectional transcription processes and the formation of double stranded RNA in the 3'UTR regions that prevent the translation should be considered, as well as the new world of non-coding RNA (ncRNA) such as microRNA, antisense RNA or long non-coding RNA (lncRNA). Neurodegenerative diseases associated with these mechanisms include myotonic dystrophy type 1 (DM1), spinocerebellar ataxia 8 (SCA8) and other types of SCA, Friedreich's ataxia (FRDA) and amyotrophic lateral sclerosis (ALS). miRNAs seem to be clearly associated also to AD and PD.
Transcriptional regulation represents another level of complexity, which opens a broader field of research ranging from the regulation (such as the expression of transcription factors or their interaction with DNA and the transcription machinery) to the attractive role of chromatin modifications and epigenetics, which could promote or not gene expression[25].
These processes have significant p-values in clusters 1, 2, 8, 10, 12 and 13.

References

1. Magistretti, P.J. & Allaman, I. *Neuron* **86**, 883–901 (2015).

2. Lourenço, C.F., Ledo, A., Dias, C., Barbosa, R.M. & Laranjinha, J. *Front. Aging Neurosci.* **7**, 103 (2015).

3. Ma, Y. & Eidelberg, D. *Mol. Imaging Biol.* **9**, 223–33 (2007).

4. Pickrell, A.M. & Youle, R.J. *Neuron* **85**, 257–273 (2015).

5. Kimura, T., Ishiguro, K. & Hisanaga, S.-I. *Front. Mol. Neurosci.* **7**, 65 (2014).

6. Kawakami, F. & Ichikawa, T. *Parkinsons. Dis.* **2015**, 734746 (2015).

7. Sepe, S., Payan-Gomez, C., Milanese, C., Hoeijmakers, J.H. & Mastroberardino, P.G. *DNA Repair (Amst).* **12**, 568–577 (2013).

8. Madabhushi, R., Pan, L. & Tsai, L.H. *Neuron* **83**, 266–282 (2014).

9. Reynolds, J.J. & Stewart, G.S. *DNA Repair (Amst).* **12**, 588–599 (2013).

10. Canugovi, C., Misiak, M., Ferrarelli, L.K., Croteau, D.L. & Bohr, V.A. *DNA Repair (Amst).* **12**, 578–587 (2013).

11. Mattson, M.P. *Nat. Rev. Mol. Cell Biol.* **1**, 120–9 (2000).

12. Alberghina, L. & Colangelo, A.M. *BMC Neurosci.* **7 Suppl 1**, S2 (2006).

13. Radi, E., Formichi, P., Battisti, C. & Federico, A. *J. Alzheimers. Dis.* **42 Suppl 3**, S125–52 (2014).

14. Ghavami, S. et al. *Prog. Neurobiol.* **112**, 24–49 (2014).

15. Yang, Y., Mufson, E.J. & Herrup, K. *J. Neurosci.* **23**, 2557–2563 (2003).

16. Bianco, M.R. et al. *J. Neurosci. Res.* **89**, 1302–15 (2011).

17. Kole, A.J., Annis, R.P. & Deshmukh, M. *Cell Death Dis.* **4**, e689 (2013).

18. Herrup, K. *Mech. Ageing Dev.* **134**, 434–439 (2013).

19. Cooper, J.D., Lindholm, D. & Sofroniew, M. V *Neuroscience* **62**, 625–629 (1994).

20. Wang, X., Huang, T., Bu, G. & Xu, H. *Mol. Neurodegener.* **9**, 31 (2014).

21. Johnson, R., Noble, W., Tartaglia, G.G. & Buckley, N.J. *Prog. Neurobiol.* **99**, 293–315 (2012).

22. Belzil, V. V., Gendron, T.F. & Petrucelli, L. *Mol. Cell. Neurosci.* **56**, 406–419 (2013).

23. Pattaroni, C. & Jacob, C. *Mol. Neurobiol.* 1–17 (2012).

24. Small, S.A. & Petsko, G.A. *Nat. Rev. Neurosci.* **16**, 126–132 (2015).

25. Renoux, A.J. & Todd, P.K. *Prog. Neurobiol.* **97**, 173–189 (2012).
