# Supplementary material for: Comparing Alzheimer’s and Parkinson’s diseases networks using graph communities structure
Source: BMC Syst Biol. 2016 Mar 2;10:25. doi: 10.1186/s12918-016-0270-7 (PMC4776441; doi:10.1186/s12918-016-0270-7)
Supplement: Additional file 3 — Pathology Specific Processes. (PDF 66 kb) [file 12918_2016_270_MOESM3_ESM.pdf]

Additional File 2 - Pathology Specific Processes

The following table lists communities of proteins that did not have any relevant counterpart in the other pathology. Thus, these processes appear to be specific of the two conditions separately. We reviewed all these processes and reported references that support these findings.

AD		PD	
**Community**	**Description**	**Community**	**Deascription**
33	Cell motility and adhesion	96	Blood vessel development
135	Lipid metabolism and transport	109	Glutamatergic synaptic transmission
163	PDGF signaling pathway	150	TGF signaling pathway
174	Tetrahydrobiopterin biosynthesis	164	Synaptic vesicles secretion
175	IGF signaling pathway	169	Dopaminergic transmission
243	IL6 and CNTF signaling pathway	179	FGF signaling pathway
330	Blood coagulation	185	Purine/pyrimidine metabolism
365	Endothelin signaling pathway	323	Chemotaxis
		364	Proteoglycan biosynthesis
		385	Inner mitochondrial membrane organization

Alzheimer's Characteristic Communities

Among biological processes that emerged as peculiar to AD development, community 33 consists of cell motility and adhesion processes, which require the intervention of transmembrane proteins and other molecular components that modify the structure of the extracellular matrix. One important member of this community is the amyloid precursor protein (APP), a transmembrane protein implicated in many neuronal processes including development, synapse formation, and neurite outgrowth; however, its amyloidogenic processing leads to extracellular β-amyloid aggregates that are one of the AD hallmarks[1–3]. Another protein that belongs to this community is thrombospondin 1, an extracellular matrix protein that, in the CNS, is predominantly produced by astrocytes and is implicated in synaptogenesis. It has been shown that accumulation of β-amyloid increases its expression, while preventing its release[4]. It is known that neuronal aggregates have both direct and indirect toxic effects by impairing synaptic functions[4,5]. As a compensatory mechanism, neurons need to modify the extracellular matrix to start synaptogenesis. Lack of thrombospondin 1 could therefore limit this process and determine neuronal dysfunction[1–5].

Community 135 includes ApoE, a protein involved in lipid transport: ApoE allele ε4 is one of the major genetic risk factors for familial AD and dementia. The precise mechanisms by which lipid-related risk factors affect the onset of AD remain to be clarified[6,7]. ApoE is mostly synthesized and secreted by astrocytes in the brain; it binds to high-density lipoproteins (HDLs) to facilitate cholesterol and phospholipids mobilization and transport by interacting with low-density lipoprotein receptor family[8]. Several hypotheses have been postulated to explain the pathological role of ApoE in the brain, including: ***i***) the amyloid scavenging properties of ApoE-HDL complexes and the removal of amyloid peptides from the brain to the cerebrospinal fluid (CSF); ***ii***) the interference with the cytoskeletal architecture and Tau functions; and ***iii***) reduced activity in the maintenance of lipid homeostasis[9–11]. Based on these observations, drugs that affect lipid metabolism or enhance ApoE activity have been tested has potential treatments for the sporadic form of AD.

There is compelling evidence that vascular dysfunction and lowered cerebral blood flow (CBF) could be key factors in AD development. In fact, most cardiovascular risk factors such as diabetes, hypertension, atherosclerosis and obesity are also risk factors for AD. The link between vascular and neurodegenerative disease is still not clear, however, AD patients have reduced CBF, and Aβ may contribute to this reduction. Several studies have demonstrated the capacity of $Aβ_{1-42}$ and $Aβ_{1-40}$ to enhance endothelium-dependent vasoconstriction *in vivo* and *in vitro* due to the direct effect of Aβ peptides on vessel smooth muscle. Cell contraction depends on the activity of endothelin-1 (ET-1), a protein synthesized and secreted as a pro-form from endothelial and neuronal cells. Palmers et al. showed overexpression of its gene (EDN-1) and protein in the neocortex in AD, a process captured by community 365[12].

In agreement with this data, community 330 concerns factors involved in blood coagulation

(coagulation factors III and VII). Recent evidence indicates a correlation between altered vascular homeostasis (FXIIIa, thrombin and fibrinogen) and the degree of AD pathology. Moreover, Aβ oligomers might influence directly or indirectly blood fluidity[13].

Community 175 includes proteins of the IGF pathway. IGF1 is a potent growth factor on somatic growth, but also an important modulator of brain function and its levels appear dysregulated in AD patients. Several studies support the ability of IGF-I to stimulate amyloid release from neurons, as well as its clearance, suggesting a role for insulin and insulin-like growth factor-I in the molecular mechanisms underlying AD pathology. Other studies reported IGFBP7 – critical regulator of memory consolidation – as a biomarker for AD, due to its up-regulation in the hippocampus of animal models of AD[14].

In addition to processes referred as characteristic of AD, we also found new hints that are yet to be elucidated. As an example, CNTF and IL-6 appear in community 243 of AD without a relevant counterpart in PD, although these molecules, IL-6 in particular, are known to be involved in neuroinflammatory responses in many brain disease[15,16]. Very few studies are also available about the relationship between AD and PDGF or collagen (community 163). Finally, to our surprise we also found that community 174 includes enzymes that catalyze the synthesis of tetrahydropterin (BH4). In addition to its role as a cofactor for the production of catecholamines mainly in dopaminergic neurons, BH4 regulates the balance of nitric oxide in endothelial cells, which is relevant to neurometabolic and neurovascular coupling. Moreover, BH4 is also an important regulator of the cellular redox state by shuttling reducing equivalents from NADPH to specific substrates[17].

Parkinson's Characteristic Communities

PD has ten specific communities, some of them representing well-established processes of this pathology, such as community 169 (related to dopaminergic transmission) and community 164 (regarding vesicles organization), which confirms the results of our approach. In details, dopaminergic neurons of *substantia nigra* project to the dorsal striatum and play a central role in the fine control of motor function. Motor dysfunction in PD is the main target of pharmacological treatments, with L-3,4-dihydroxyphenylalanine (L-DOPA) being the most effective drug. A broad list of pharmacological agents also exists, which includes inhibitors of L-aromatic amino acid decarboxylase enzymes, monoamine oxidase (MAO) inhibitors and catechol-O-methyl transferase (COMT) inhibitors. Several dopaminergic agonists are also available, as well as anticholinergic drugs. Furthermore, it is well established the role of oxidative stress in the progression of PD and one possible mechanism responsible for the increase of oxidative stress in DAergic neurons involves the redox reactions specific to DA. In fact, at physiological pH cytosolic DA can self-oxidize to form reactive oxygen species. Moreover, DA is degraded by MAO that catalyzes its oxidative deamination leading to the production of hydrogen peroxide and 3,4-dihydroxyphenylacetaldehyde (DOPAL)[18,19].

Community 164 contains proteins implicated in vesicle transport of neurotransmitters. Lewy’s body are pathologically characterized by the accumulation of the presynaptic protein a-synuclein. Although the physiological function of this protein is not fully elucidated, its localization with DA vesicles and and loss of dopaminergic transmission (as a result of its overexpression), suggest that α-synuclein is implicated in the regulation of neurotransmitter release, in particular in vesicle storage of DA. Moreover, α-synuclein seems to interact with proteins of RAB small GTPase family and act as a chaperone protein for SNARE proteins[20–22].

DA is not the only neurotransmitter implicated in PD pathogenesis. In fact, glutamate (community 109) also plays a central role in excitatory transmission in basal ganglia. Therefore, alteration of glutamate homeostasis might have a significant impact on neurons through excitotoxicity cascades. DAergic projections from *substantia nigra* to basal ganglia exert complex changes in the glutamatergic pathways. It has been hypothesized that in PD a compensatory mechanism aims to increase the release of glutamate onto DAergic neurons located in *substantia nigra* and consequent release of DA to maintain DA homeostasis. However, a sustained increase in glutamate could elicit

an excitotoxicity cascade and augment neurodegeneration. Evidence of compensatory mechanisms via glutamate has been found not only in pathological state of DAergic circuit, but also in altered cholinergic projections in PD[23–25].

Community 96 contains components for “blood vessels development” which are relevant for re-organization of the extracellular matrix during angiogenesis, such as matrix metalloproteinase 2 (MMP2)[26]. However, mechanisms involving MMPs can be also found in AD map, based on the increasing evidence of MMPs in Aβ catabolism and clearance, as well as in the degradation of Nerve Growth Factor, a process linked to reactive astrogliosis[27]. Related to the extracellular matrix, community 364 also includes enzymes involved in the biosynthesis of proteoglycans and other molecules of the extracellular matrix.

In the community 385, we found molecular components of the mitochondrial import machinery, such as TIMM9 and TIMM10. Although the role of mitochondrial dysfunction in the onset and progression of neurodegenerative diseases is well known, this kind of computational analysis has not highlighted any other community whose terms are related to mitochondrial processes. Several studies have demonstrated a link between mitochondrial accumulation of APP and α-synuclein and the pathogenesis of AD and PD[28]. In fact, both proteins contain signal sequences for their mitochondrial trafficking, therefore there should be an interaction between APP or α-synuclein and the import receptor complex on the inner membrane.

Interestingly community 323 contains chemokines, small proteins released during the inflammatory response to recruit cells of the immune system or implicated in the control of cell migration during tissue development. Chemokines play a role in the physiology of the nervous system, including neuronal migration, cell proliferation and synaptic activity. Although this community was found as specific for PD, significant differences of chemokines and their receptors level in serum, cerebrospinal fluid (CSF) and brain tissue have been found in AD patients and normal people. Under pathological conditions, chemokines act as chemoattractants to guide cell migration, thus regulating the migration of microglia, astrocytes, neurons and neural progenitors to neuroinflammation sites caused by senile plaques of amyloid[29].

In the community 150, we found proteins involved in TGFβ pathway. TGFβ has been implicated in a broad range of biological activities, including extracellular matrix synthesis, inhibition and stimulation of cell proliferation, inhibition and stimulation of inflammatory cell infiltration, immunosuppression, chemoprotection, and neuroprotection. TGFβ has also been shown to play a role in several CNS pathologies including PD, AD and ischemia. All TGFβ isoforms also provided potent neuroprotection against MPTP and MPP^+, two compounds that exhibit selective neurotoxicity on DAergic neurons, therefore used in cellular and animal of PD[30]. Another growth factor important in the development of the nervous system is FGF, captured in community 179, at the moment only marginally involved in neurodegenerative diseases. Finally, it remains very elusive the relevance of community 185, whose terms are related to the biosynthesis of purine and pyrimidine.

References

1. Morgan, C., Colombres, M., Nuñez, M.T. & Inestrosa, N.C. *Prog. Neurobiol.* **74**, 323–49 (2004).

2. Perez, R.G., Zheng, H., Van der Ploeg, L.H. & Koo, E.H. *J. Neurosci.* **17**, 9407–14 (1997).

3. Chapman, P.F. et al. *Nat. Neurosci.* **2**, 271–6 (1999).

4. Rama Rao, K. V, Curtis, K.M., Johnstone, J.T. & Norenberg, M.D. *J. Neuropathol. Exp. Neurol.* **72**, 735–44 (2013).

5. Yatin, S.M., Varadarajan, S., Link, C.D. & Butterfield, D.A. *Neurobiol. Aging* **20**, 325–30; discussion 339–42

6. Florent-Béchard, S. et al. *Biochimie* **91**, 804–9 (2009).

7. Poirier, J. et al. *Neurobiol. Aging* **35 Suppl 2**, S3–10 (2014).

8. Beffert, U., Stolt, P.C. & Herz, J. *J. Lipid Res.* **45**, 403–9 (2004).

9. Kim, J., Basak, J.M. & Holtzman, D.M. *Neuron* **63**, 287–303 (2009).

10. Brecht, W.J. et al. *J. Neurosci.* **24**, 2527–34 (2004).

11. Poirier, J. *Neurobiol. Aging* **26**, 355–61 (2005).

12. Palmer, J. & Love, S. *Pharmacol. Res.* **63**, 525–31 (2011).

13. Zainaghi, I.A., Forlenza, O. V & Gattaz, W.F. *Psychopharmacology (Berl).* **192**, 547–53 (2007).

14. Agbemenyah, H.Y., Agis-Balboa, R.C., Burkhardt, S., Delalle, I. & Fischer, A. *Neurobiol. Dis.* **62**, 135–43 (2014).

15. Erta, M., Quintana, A. & Hidalgo, J. *Int. J. Biol. Sci.* **8**, 1254–66 (2012).

16. Garcia, P. et al. *J. Neurosci.* **30**, 7516–27 (2010).

17. Longo, N. *J. Inherit. Metab. Dis.* **32**, 333–42 (2009).

18. Meiser, J., Weindl, D. & Hiller, K. *Cell Commun. Signal.* **11**, 34 (2013).

19. Bisaglia, M., Greggio, E., Beltramini, M. & Bubacco, L. *FASEB J.* **27**, 2101–10 (2013).

20. Lashuel, H.A., Overk, C.R., Oueslati, A. & Masliah, E. *Nat. Rev. Neurosci.* **14**, 38–48 (2013).

21. Alter, S.P., Lenzi, G.M., Bernstein, A.I. & Miller, G.W. *Curr. Neurol. Neurosci. Rep.* **13**, 362 (2013).

22. Eisbach, S.E. & Outeiro, T.F. *J. Mol. Med. (Berl).* **91**, 693–703 (2013).

23. Caudle, W.M. & Zhang, J. *Exp. Neurol.* **220**, 230–3 (2009).

24. Gardoni, F. & Bellone, C. *Front. Cell. Neurosci.* **9**, 25 (2015).

25. Mehta, A., Prabhakar, M., Kumar, P., Deshmukh, R. & Sharma, P.L. *Eur. J. Pharmacol.* **698**, 6–18 (2013).

26. Lorenzl, S., Albers, D.S., Narr, S., Chirichigno, J. & Beal, M.F. *Exp. Neurol.* **178**, 13–20 (2002).

27. Colangelo, A.M., Cirillo, G., Lavitrano, M.L., Alberghina, L. & Papa, M. *Biotechnol. Adv.* **30**, 261–71

28. Schon, E.A. & Przedborski, S. *Neuron* **70**, 1033–53 (2011).

29. Doty, K.R., Guillot-Sestier, M.-V. & Town, T. *Brain Res.* **1617**, 155–73 (2014).

30. Pratt, B.M. & McPherson, J.M. *Cytokine Growth Factor Rev.* **8**, 267–92 (1997).
